# Role of ADAM10 as a CD30 Sheddase in Classical Hodgkin Lymphoma

**DOI:** 10.3389/fimmu.2020.00398

**Published:** 2020-03-31

**Authors:** Hinrich P. Hansen, Adriana F. Paes Leme, Michael Hallek

**Affiliations:** ^1^Department I of Internal Medicine, Center for Integrated Oncology Aachen Bonn Cologne Düsseldorf, Center for Molecular Medicine Cologne, CECAD Center of Excellence on Cellular Stress Responses in Aging-Associated Diseases, University Hospital of Cologne, Cologne, Germany; ^2^Laboratório de Espectrometria de Massas, Laboratório Nacional de Biociências, Centro Nacional de Pesquisa em Energia e Materiais, Campinas, Brazil

**Keywords:** Hodgkin lymphoma, CD30, ADAM10, antibody-drug conjugate, extracellular vesicle, cancer treatment

## Abstract

Cancer cells generally recruit and influence non-malignant immune cells to support the tumor growth. Classical Hodgkin lymphoma (cHL) is a good example because the affected lymphoid tissue contains only a few malignant Hodgkin and Reed-Sternberg (H-RS) cells, which are supported by a massive infiltrate of lymphocytes, fibroblasts, and innate immune cells. The transmembrane receptor CD30, which is selectively expressed on the H-RS cells, plays an important role, not only in cell stimulation and intercellular communication but also in tumor diagnosis and targeted tumor therapy. Different protein processing pathways influence its functionality. Depending on the conditions, the receptor is internalized or released. The release of CD30 occurs either as an intact molecule, embedded in the membrane of extracellular vesicles (EVs), or as a cleaved soluble ectodomain (sCD30). CD30 cleavage is predominantly catalyzed by ADAM10. The enzyme is catalytically active in cells as well as in EVs and gradually releases sCD30. Because the circulation contains no CD30^+^ donor cells, this mechanism explains that the cleaved ectodomain represents the predominant form of CD30 in the plasma of cHL patients. CD30 processing might influence the impact of CD30 antibody-drug conjugates, such as Brentuximab Vedotin (BV). Whereas, ADAM10-degraded CD30 impedes the BV efficacy, tumor-derived EVs load bystander cells with CD30 and generate new targets among supporter cells. This crossfire effect might contribute to the enormous clinical impact of BV, whereas the ADAM10-dependent cleavage to the mild systemic off-target effects of the treatment with BV.

## Introduction

Classical Hodgkin lymphoma (cHL) is a malignant disease with a low percentage (~1%) of malignant cells and a huge proinflammatory infiltrate in the affected tissue (1). The crosstalk between tumor and bystander cells plays a critical role in the development and maintenance of the disease (2). Therefore, the tumor cells influence bystander cells through direct cell contact and release of soluble mediators to support the tumor cell survival (3). CD30 is a receptor of the TNF receptor superfamily (TNFRSF8), and selectively expressed on the malignant cells in cHL. However, CD30 is also released, either on extracellular vesicles (EVs) or as a truncated ectodomain (sCD30) after cleavage by A Disintegrin And Metalloproteinase 10 (ADAM10). In this article, we review the role of ADAM10 in the release of CD30 and the consequences for the CD30 functionality in cell communication and immunotherapy.

## Hodgkin Lymphoma

The malignant mononucleated Hodgkin (H) and multinucleated Reed-Sternberg (RS) cells release diverse cytokines and chemokines to attract and to communicate with the proinflammatory cells of the environment, including T and B cells, plasma cells, macrophages, dendritic cells, neutrophils, eosinophils, mast cells, and fibroblasts (3). This Hodgkin-typical microenvironment contributes to the maintenance of the disease and drug resistance (2). The crosstalk between H-RS and bystander cells involves multiple interactions through direct cell contact and the release of signaling molecules (1). In particular, the mast cell infiltration correlates with a worse disease-free survival (4) and a high eosinophil count is a marker for a poor prognosis (5). In cHL, mast cells and eosinophils typically express the membrane-anchored CD30 ligand (CD30L/TNFSF8). However, the CD30 receptor is restricted to H-RS cells and a small population of non-malignant activated B- and T blasts (6). The specific ligation of CD30 leads to reverse signaling in bystander cells (7). In mast cells, which contribute to 66% of CD30L^+^ cells in cHL, the CD30L crosslinking causes a degranulation-independent release of cHL-typical proinflammatory cytokines, such as IL-8, CCL3, and CCL4 (8). However, the CD30L^+^ bystander cells are rarely found in the vicinity of the H-RS cell, which raised the question if soluble forms of CD30 participate in the CD30-CD30L crosstalk over distance.

## Release of Membrane-Proteins—a Complex Scenario

There are two principle mechanisms how membrane proteins can influence distant cells. (i) Cells can release membrane-enclosed vesicles, which carry proteins, lipids, and nucleic acids as a payload of the donor cell (9). They transport membrane proteins in the natural orientation and in a plasma membrane context and are able to mimic a complex cell-cell interaction distant from the donor cell. (ii) The ectodomains of transmembrane proteins can be cleaved close to the lipid bilayer and subsequently released as soluble molecules. In most cases, this ectodomain shedding is catalyzed by members of the ADAM family such as ADAM10 or ADAM17. Both are transmembrane proteins, which exert protease activity through a Zn^2+^-dependent catalytic domain on a wide panel of membrane proteins (10). In premature stages, they are blocked by an inhibitory prodomain. Mature ADAM10 and typical ADAM substrates, including CD30, are also found in EVs. Therefore, it is possible that ectodomain shedding also occurs in EVs.

## Extracellular Vesicles from cHL Cells

Healthy cells release vesicles that are derived from multivesicular bodies (MVBs) or bud from the plasma membrane (11). Because the vesicle types have an overlapping diameter, functionality and marker proteins, the International Society of Extracellular Vesicles (ISEV) suggests to collectively refer to them as extracellular vesicles (EVs) (12). Electron microscopy, nanoparticle tracking analysis (NTA), and mass spectrometry revealed that the EVs from Hodgkin cells have an EV-typical diameter between 40 and 800 nm (mean around 140 nm) and they express CD30 and ADAM10 (13, 14). They also show a strong exposure of the lipid phosphatidylserine (PS) on the outer membrane leaflet (13). In quiescent cells, PS is restricted to the inner membrane leaflet. However, upon stimulation, a certain amount of PS flips to the outer leaflet, where the negatively charged PS interacts with the basic membrane-proximal domain (MPD) of ADAM10 and 17 and serves as a sheddase-activating trigger (15). Therefore, ADAM10-dependent ectodomain shedding might also occur in EVs from Hodgkin cells.

## ADAM-Dependent CD30 Shedding in Cells

CD30 can be cleaved by ADAM10 or ADAM17 (10). Cell stimulation influences the extent of cleavage and the selection of the sheddases. Experiments with ADAM10 or ADAM17-defective mouse embryonal fibroblasts (MEFs) and the application of wide-spectrum (BB-3644 and GW280264X) and a preferential ADAM10 (GI254023X) metalloproteinase inhibitor suggest that activation by phorbol myristate acetate (PMA) and radical oxygen species (ROS) production by cytotoxic drugs favor ADAM17-dependent cleavage of CD30 (16, 17). However, the constitutive and the CD30L-dependent cleavage is predominantly caused by ADAM10.

## Quantification of Released CD30 Forms

Both uncleaved EV-associated CD30 and sCD30 are found in the cell supernatant of cHL cells, but only EV-associated CD30 functionally influences CD30L^+^ bystander cells to release IL-8 (13). *In vitro*, only a minority of released CD30 is EV-associated, ranging between 1.7 and 3.7% in the supernatants (SN) of L428 and L1236 cells, respectively (14). *In situ*, the tumor microenvironment might modify this ratio. Particularly, fibroblasts of the nodular sclerosing subtype of cHL express high amounts of the tissue inhibitor of metalloproteinases 3 (TIMP3) (18). Although TIMP3 principally inhibits ADAM10 and ADAM17, it predominantly blocks ADAM10 *in vivo* (19). Thus, sheddase inhibition in the tumor microenvironment of certain cases of cHL might influence the amount of EV-associated CD30.

## Ectodomain Shedding of CD30 on EVs

Not many reports demonstrate that ADAM10 cleaves membrane proteins in EVs. One example is the cleavage of CD44 and L1 on EVs from ovarian carcinoma cells (20). Isolated EVs from cHL cells degrade the artificial ADAM10-selective substrate PEPMCA001 and CD30, the latter resulting in a CD30 reduction to 71% of the inhibited control after 18 h of incubation. These data indicate that CD30 is slowly cleaved on isolated EVs (14). *In situ*, there might be another kinetic of CD30 cleavage because natural inhibitors and additional enzymes might influence CD30 shedding. Thus, EV-associated ADAM10 from other cells might participate in CD30 cleavage (21). Nevertheless, CD30 is not completely cleaved when EVs harbor in the circulation, because in the blood of cHL patients, a low percentage of CD30 is EV-associated (14).

## CD30 Shedding on EVs Alters its Functionality in Targeted Immunotherapy

**Figure 1:**
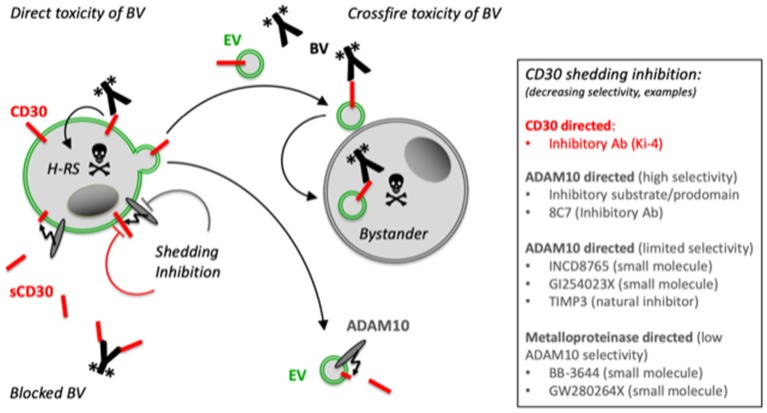
Proposed model for the role of EVs and CD30 shedding for immunotargeting with BV. The malignant H-RS cells selectively express CD30. The CD30 ADC BV binds to CD30^+^ tumor cells, is internalized and the cytotoxic compound monomethyl auristatin E (MMAE) is cleaved and activated by lysosomal proteases. H-RS cells also release CD30 on EVs. Such EVs also bind BV and target typical bystander cells such as mast cells or eosinophils. Both, the H-RS cells and the EVs also express the CD30 sheddase ADAM10, which gradually cleaves CD30 and releases sCD30. This cleavage of CD30 on cells and generation of competitive sCD30 might impair the direct efficacy of BV and the loss of CD30 on EVs might limit the crossfire functionality of EVs in the tumor microenvironment. Selective CD30 shedding inhibitors might be promising cotherapeutic drugs to improve the efficacy of CD30-based immune therapeutics with manageable off-target effects. *Indicates the toxic monomethyl auristatin A (MMAE) of BV. *Indicates the toxic monomethyl auristatin E (MMAE) of BV.

Brentuximab Vedotin (BV) is an effective CD30-directed antibody-drug conjugate (ADC) for the treatment of patients with CD30^+^ lymphomas, which are refractory to standard therapy (22). Surprisingly, this ADC is also effective in cases of diffuse large B-cell lymphoma (DLBCL) without CD30^+^ tumor cells, provided CD30^+^ bystander cells can be detected (23). Inversely, eosinophils, which are typical bystander cells in cHL, bind CD30^+^ EVs and the coapplication of the ADC BV causes cell damage also in CD30^−^ eosinophilic cells. Here, the effect depends on the presence of BV and CD30^+^ EVs (Figure 1) (14). By contrast, the coincubation of the same CD30 concentration of sCD30 was almost ineffective. Thus, CD30 ectodomain cleavage might not only result in an irreversible change of the functionality of CD30 in intercellular signaling but also in targeted immunotherapy.

## Conclusions and Outlook

CD30 is selectively expressed on H-RS cells in cHL and released in EVs or shed by the action of ADAM metalloproteinases, predominantly ADAM10 (Figure 1). However, only EV-associated CD30 functionally communicates with CD30L^+^ supporter cells. EV-associated CD30 might contribute to the effective treatment of cHL patients with the anti-CD30 ADC BV because BV damages not only CD30^+^ tumor cells but also bystander cells, when they are loaded with CD30^+^ EVs. However, this crossfire effect is limited since CD30 is gradually depleted on EVs by ADAM10 and the resulting sCD30 cannot help to damage bystander cells with BV. By contrast, high levels of sCD30 are suspected to mitigate anti-CD30 therapeutic strategies, also including CD30-directed chimeric antigen receptor T (CAR-T) cells (24). Surprisingly, an earlier study claimed that sCD30 does not significantly hamper the antitumor efficacy of CD30 CAR-T cells, maybe because effective CD30 CAR-T cell therapy needs a high CD30 density on target cells (25, 26). Another explanation is that monovalent sCD30 binds with lower avidity than the multiple binding sites of the target cells (14).

Nevertheless, CD30 shedding inhibition might improve the efficacy of CD30-directed therapeutics. The hydroxamic acid-based inhibitor BB-3644 strongly reduced sCD30 concentrations in the blood of animals with cHL xenotransplants (27). However, BB-3644 inhibits different metalloproteinases and this might have been a reason why a clinical phase I dose-escalation study with BB-3644 showed dose-limiting musculoskeletal toxicities and was discontinued (28). More selective ADAM10 inhibitors, such as the synthetic LT4 and INCD8765, the ADAM10 antibody 8C7, and an exocite-binding artificial substrate might provide a better alternative with less side effects (Figure 1) (21, 29, 30).

Still, ADAM10 accounts for the cleavage of diverse substrates, such as cadherins, membrane-spanning receptors, and ligands, and conditional knockout studies revealed an important role of ADAM10 in the functionality of endothelial cells, keratinocytes, neuron, and immune cells (31). Therefore, a substrate-selective shedding inhibitor can be favorable in a clinical setting. Humanized fragments of the shedding-inhibiting CD30 antibodies Ki-4 and Ber-H2 are possible candidates (32). These antibodies belong to a serological cluster of CD30 antibodies that does not compete with C10, the backbone antibody of BV. In conclusion, ADAM10 strongly influences the posttranslational fate and functionality of CD30. However, ADAM10 inhibition in cHL can be a double-edged sword because it might not only improve the therapy with anti-CD30 therapeutics but also favor the tumor-supporting, EV-based communication in cHL tissue.

## Author Contributions

HH, AP, and MH wrote and read the manuscript.

### Conflict of Interest

The authors declare that the research was conducted in the absence of any commercial or financial relationships that could be construed as a potential conflict of interest.
